# A study on community older people’s willingness to use smart home—an extended technology acceptance model with intergenerational relationships

**DOI:** 10.3389/fpubh.2023.1139667

**Published:** 2023-06-06

**Authors:** Wenjing Wei, Xiaodong Gong, Jian Li, Kun Tian, Kai Xing

**Affiliations:** ^1^School of Animation and Digital Media Arts, Communication University of Shanxi, Jinzhong, Shanxi, China; ^2^School of Design and Arts, Beijing Institute of Technology, Beijing, China

**Keywords:** older adult, smart-home services, technology acceptance model, intergenerational support, family

## Abstract

**Introduction:**

Despite the potential of smart home technology to promote sustainable lifestyles, the adoption rate among older adults remains relatively low. This study aims to investigate the influence of intergenerational relationships on the acceptance of smart home services among seniors.

**Methods:**

A survey was conducted among 298 older adults in China, and data were analyzed using Partial Least Squares Structural Equation Modeling (PLS-SEM). Ten predictor variables were examined to assess their impact on the willingness to use smart home services.

**Results:**

Intergenerational relationships significantly influenced the utilization of smart home services among older adults. Specifically, intergenerational instrumental support had a direct positive effect on the behavioral intention to use smart homes. Additionally, intergenerational emotional and financial support affected life satisfaction, which subsequently influenced the behavioral intention to use smart homes.

**Discussion:**

The assistance and guidance provided by younger generations play a crucial role in shaping the willingness of older adults to adopt smart home technology. Intergenerational support can contribute positively to enabling aging individuals to age in place through the utilization of technology.

## Introduction

In recent years, there has been a significant increase in the older adult population in China (1), which has resulted in new challenges for social retirement. It is essential to note that the majority of seniors desire to live independently for as long as possible to increase their satisfaction and prevent them from incurring costly institutional care. Smart-home services for the older adult focus on providing better aging-in-place services to promote a happier life.

Initially, smart home technologies were developed with a focus on security and energy efficiency (2). As time went by, the range of users gradually expanded to include vulnerable individuals such as the older adult, and people with chronic diseases (3, 4). Smart home services are home automation services based on Internet of Things (IoT) technologies that can be purchased, prefabricated, or installed at home (5). These services include security systems, keyless entry, body detection devices, smart lighting, smart water valves, and more. In the past decade, various smart home services have been utilized to address the unique needs of in-home aging. Numerous studies have shown that smart home services are beneficial to the well-being of the older adult (6). The benefits of smart home services include independent living, improved healthcare, social involvement, safety, cost reduction, and decision making (7). Therefore, smart home services can help maintain independence and improve the quality of life (8). Scholars have proposed various flexible smart-home service designs to address the specific needs of the older adult (9, 10).

Although technology advancements bring apparent benefits, promoting even the best smart home devices may face potential obstacles such as high expense, technological challenges, safety concerns, burden on others, difficulty in recalling functions, stigmatization, and lack of perceived need (6, 11, 12). Consequently, the older adult do not use them. In China, the adoption rate of smart homes among the older adult remains low (13).

We all know that modern seniors are not entirely resistant to new technologies. They can adopt new technology under the influence of various factors and may even actively try new products. Numerous scholars are investigating the positive attitudes of older adults toward smart-home services and the factors that influence these attitudes. Identifying these positive factors can effectively promote the use of more smart-home services among senior citizens, which can ultimately have a substantial impact on their well-being.

## Related works

The Technology Acceptance Model (TAM) is inadequate for assessing the primary factors and barriers to the adoption of smart home services by older adult individuals. To address this issue, Chen and Chan (14) proposed the Senior Technology Acceptance Model (STAM) specifically for the older adult population in Hong Kong, while Pal et al. (15) developed the Older Adult Smart Home Technology Acceptance Model (ESHTAM), a comprehensive model for smart home technology acceptance among older adult individuals. Several studies have examined the factors that influence the acceptance of smart home services by older adults, including personal and environmental perception factors. Personal perception factors comprise perceived ease of use, perceived usefulness, perceived cost (16), facilitating conditions (7),technology anxiety (17), and security and privacy concerns (16–18). Environmental perception factors include social influence (7, 18), subjective norms (15), cultural influence (7), family management policies, and government policies (18).

A literature review conducted by Peek et al. (19) revealed a dearth of valid quantitative studies during the post-implementation phase of technology adoption by older adults. Tsertsidis et al. (20) discovered that the perceptions of older adults regarding technology changed between the pre-implementation and post-implementation stages in their investigation of smart home technology acceptance. They observed that some of the negative concerns expressed during the pre-implementation stage were viewed positively during the post-implementation stage. Similarly, Ghorayeb et al. (11) reported that older adult consumers’ approval of smart home monitoring equipment increased with use, in contrast to those who had not utilized monitoring technology. The factors influencing technology adoption also vary across different stages of use in old age. For instance, the longer an older adult individual uses technology, the greater the influence of social factors on their technology adoption behavior (21). Therefore, it is important to focus on changes in the factors affecting user acceptance attitudes after technology implementation when studying the intention to use smart homes.

Research on technology adoption by the older adult has been criticized for excessively focusing on models such as TAM and UTAUT (Unified Theory of Acceptance and Use of Technology) while ignoring other critical variables. For instance, Chen and Chan’s (22) literature review found that despite unique psychosocial factors of the older adult, such factors were often disregarded. Such psychosocial factors may arise from their changing intergenerational relationships.

With the development of technology and the popularization of digitalization, intergenerational interaction and communication have become more widespread and frequent, which also affects intergenerational support (23). Intergenerational support is defined as “the process of economic reciprocity, mutual assistance, and emotional support between generations in a family, as well as the sharing of life experiences and resources” (24). It is a two-way exchange behavior between generations. Studies in developed Western countries have shown that intergenerational support flows from older adults to their offspring. However, research conducted in developing countries indicates that intergenerational support primarily flows from younger to older generations (25, 26). This difference may be due to the fact that in developing countries, older adult care is primarily funded by households rather than the government. Intergenerational support is a broad concept and can be classified into instrumental, financial, and emotional support (27, 28).

Intergenerational support has a significant impact on many aspects of older adults’ lives. In general, family support exchange, regardless of the type of support, has a positive effect on the lives of older adults and positively impacts their mental health, self-esteem, and well-being (29–31). Both emotional and financial support enhance physical and mental health (32). There have also been numerous studies on the effect of intergenerational support on technology acceptance among older adults. Seniors who lack daily exposure to new technology are more likely to develop mistrust and negative attitudes towards technological products and ultimately resist adopting new technologies (33). Family structure and communication provide an opportunity to overcome these attitudes barriers. Positive intergenerational interactions can encourage older adults to become interested in ICT (information and communications technology) and start learning about technology, ultimately having a positive external effect on their adoption of new technological items (34, 35). Some studies have shown that the number of minors in the home is positively associated with the willingness of older adults to use Internet technology, although there is no significant effect on the intensity of use (36). However, other studies have found that the presence of children does not clearly explain the adoption of Internet technology by their parents and may be due to the Internet demands associated with having children in the home (37). Further research is needed on the specific effects of intergenerational relationships in families.

Intergenerational support can affect older adults’ technology acceptance in several ways. The current focus is on intergenerational technical support provided by children (or grandchildren) to older adults. Grandchildren can encourage grandparents to adopt new technologies by demonstrating and explaining how to use various devices (38). Research has confirmed that the involvement of young “enthusiastic experts” can facilitate intergenerational knowledge transfer and ultimately promote the use of new technologies by older adult users (36, 39). “Enthusiastic experts” provide both intergenerational instrumental and emotional support. He and Huang (40) found that intergenerational technological support has a positive effect on seniors’ attitudes towards smartphone use and their well-being. However, assistance from family members (or intergenerational home care) may act as a substitute for technology, negatively affecting older adults’ acceptance of home technology (41, 42), indicating the need for further research. Several studies have examined seniors’ adoption of digital feedback (bottom-up technology transfer). Factors that facilitate the acceptance of digital feedback from younger generations by older adult individuals include lower age, higher literacy level, higher economic status, and good family communication practices (43).

Intergenerational support can have both positive and negative effects on older adults’ acceptance of smart home services. However, there is a lack of in-depth quantitative studies in this area. The attitudes of older adults towards the use of new technology devices and the type of intergenerational support they receive are unclear, and no research has examined the effect of specific types of intergenerational support on technology acceptance by older adults. There is also a lack of research on the impact of intergenerational financial support. These challenges suggest that intergenerational support should be incorporated into the concept of senior acceptance of smart home services. More quantitative research is needed to determine which factors are most influential. Therefore, this study aims to integrate the TAM model with intergenerational support theory and investigate the influence of three major factors on the willingness of older adults to use smart home services.

## Research model and hypothesis

Davis et al. (44) proposed the Technology Acceptance Model (TAM), which has been extensively used as a theoretical model to investigate the intention to use different ICT technologies and intelligent systems. In this study, a TAM-based research model is proposed to investigate the effectiveness of smart home services for older adults.

### Perceived usefulness, perceived ease of use, and intention to use

The Technology Acceptance Model (TAM) framework comprises of Perceived Usefulness (PU), Perceived Ease of Use (PEOU), and Behavioral Intention (BI). According to TAM, PEOU refers to “the effortlessness experienced by older adults while using smart-home services,” whereas PU pertains to “the extent to which older adults believe that smart-home services can enhance their overall quality of life.” Previous studies have shown that both PU and PEOU significantly affect the intention of older adults to use smart-home services, either directly or indirectly through attitudes (16, 45).

European and American researchers concur that the effects of PU and PEOU differ significantly between pre- and post-implementation stages (20, 46). Similar conclusions were reached in studies conducted in Asia. For example, when mature Asian users over the age of 40 use the Internet, the impact of usefulness becomes weaker during the initial Internet adoption phase, as compared to the impact of perceived ease of use (47).

The stage of our current study is the initial adoption stage, and therefore, based on the above discussion, we hypothesize:

*H1:* PEOU has a significant positive effect on the PU of the services.

*H2:* PU has a positive effect on the BI to use smart-home services.

*H3:* PEOU has a significant positive effect on the BI to use smart-home services.

### Life satisfaction

Life satisfaction is a crucial aspect of well-being, which is a prerequisite for successful aging (48). Previous research has shown that an active lifestyle and participation in social activities can increase the willingness to learn and adopt new technological advancements (49). Moreover, Chen and Chan (14) found that life satisfaction can significantly influence technology use behavior.

*H4:* Life satisfaction positively influences BI to use smart-home services.

### Intergenerational instrumental support

The concept of intergenerational instrumental support is broad and typically includes practical or tangible forms of support, such as household chores and personal care (50). However, in this paper, intergenerational instrumental support is defined in digital technology-related aspects, which are crucial as technology advances and older adults face a “digital divide” due to a lack of digital skills. In response, children and grandchildren in the family may become “passionate experts” who provide digital products to older adults and support them in learning to use various IT products. The intergenerational instrumental support studied in this research includes “children providing intelligent products to older adults along with bottom-up technology transfer” (51). Unlike traditional studies, this work incorporates intergenerational technology support into intergenerational instrumental support, and focuses on the impact of “upward” intergenerational support on BI, whereby older adults are the primary recipients of intergenerational instrumental support rather than providers.

Cao et al.’s (52) study revealed that intergenerational instrumental support not only directly reduces older adult users’ resistance behavior towards mHealth application, but also mitigates the impact of negative emotions on resistance behavior. Meanwhile, Eynon and Helsper (39) argued that having children in the household increases the number of Internet accesses for various purposes but does not improve adults’ confidence and skills in using the Internet.

Based on the preceding discussion, we hypothesize that the more intergenerational instrumental support older adults receive for information technology, the more likely they are to accept and utilize smart home services. Therefore, we propose that:

*H5:* Intergenerational instrumental support has a significant positive effect on the PU of the services.

*H6:* Intergenerational instrumental support has a significant positive effect on the BI to use smart-home services.

### Intergenerational emotional support

Intergenerational emotional support is a crucial factor in assessing emotional cohesion between parents and children. Research has shown that older adults who receive emotional support from their children and reciprocate the support are more likely to have higher levels of life satisfaction (53, 54), mental health (55, 56) and well-being (57). For instance, a study by Lai et al. (58) revealed that among older adult Chinese immigrants living in the United States, having closer relationships with grandchildren significantly improved their self-reported quality of life. As quality of life is closely related to life satisfaction (48), this finding highlights the importance of intergenerational emotional support for older adults’ well-being.

Moreover, Zhou and Ding (59) found that women who had closer family ties were more likely to receive digital product recommendations and digital technology education from their children. This result is consistent with other studies that have demonstrated how family harmony can enhance digital feedback (60, 61). Thus, it is reasonable to argue that intergenerational emotional support has a positive impact on intergenerational instrumental support.

Chen and Chan (14) investigated the patterns of ICT usage among senior citizens in Hong Kong. They found that older adults who are socially well-connected were more likely to intend to use such technologies, possibly due to receiving more family support. We hypothesize:

*H7:* Intergenerational emotional support positively affects older adults’ life satisfaction.

*H8:* Intergenerational emotional support positively influences BI to use smart-home services.

*H9:* Intergenerational emotional support positively influences intergenerational instrumental support.

### Intergenerational financial support

Intergenerational financial support includes both downward and upward monetary support. However, studies in China have shown that the older adult are the main recipients of intergenerational financial support, which is the opposite of what is seen in Western countries (25). This difference may be attributed to economic growth and a cultural emphasis on filial piety. Intergenerational financial support has an impact on the psychological well-being of older adults. Numerous studies have shown that higher levels of financial support for older adults are associated with improved psychological well-being and greater life satisfaction (55, 62, 63). Based on these discussions, we hypothesize:

*H10:* Intergenerational financial support positively affects older adults’ life satisfaction.

### Demographic data

In the older adult population of Hong Kong, age and gender directly influence the use of geriatric technology (14). In the field of smart homes, Arar et al. (17) reported that age is the most significant determinant of the acceptability of smart home services among the older adult in the UAE. So, the experiment’s control variables include age and gender.

**Figure 1:**
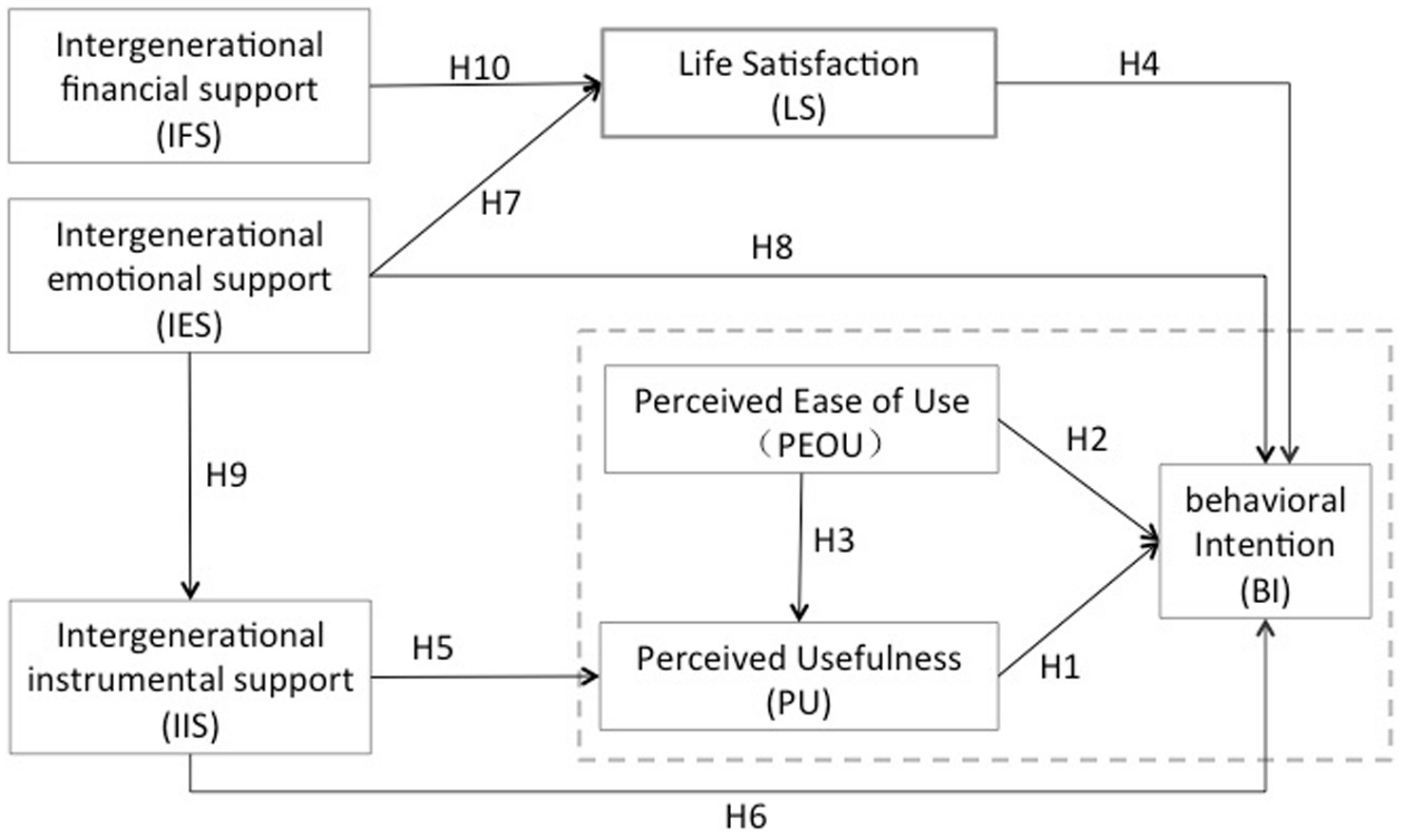
Theoretical framework and hypotheses. Control variables: gender, age.

Our study aims to investigate the impact of these variables on the acceptance of smart home services among the older adult. Figure 1 displays the specific relationships among the variables based on the research hypotheses mentioned above.

## Experimental process

**Figure 2:**
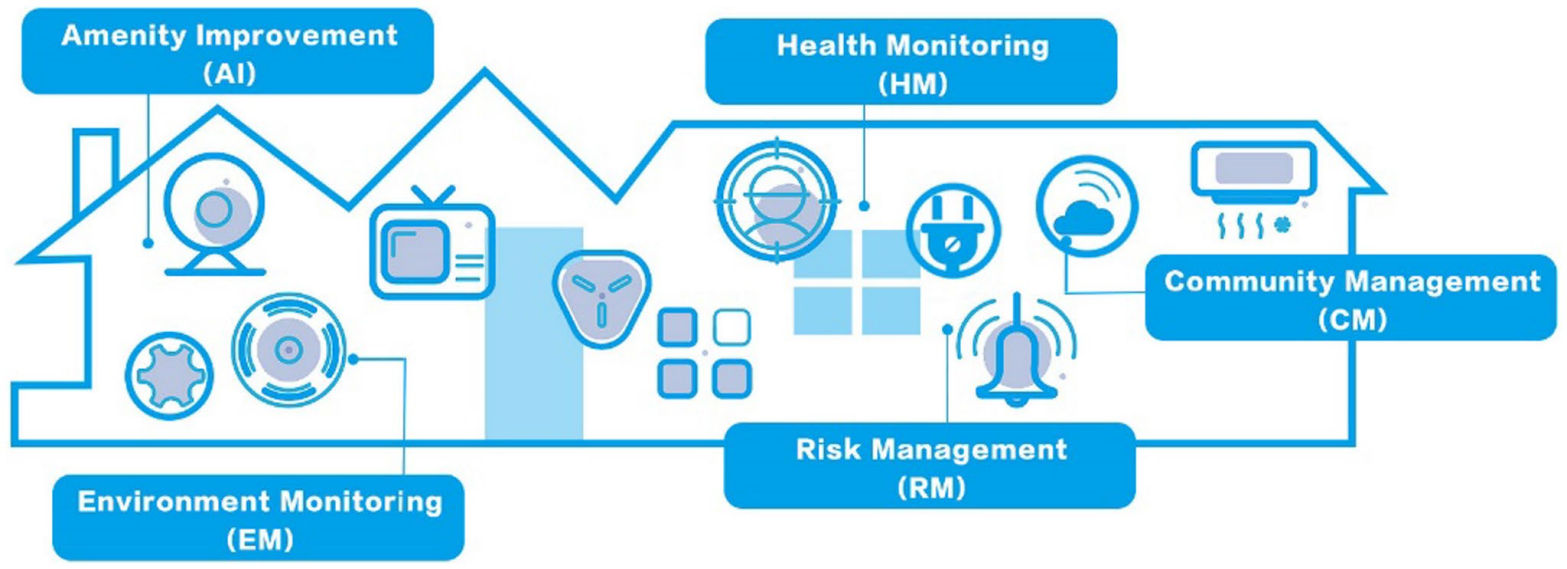
A universal model of smart home services that meets the well-being of the older adult.

We have developed an online survey tool to measure the perceptions and intergenerational support of the older adult for smart home services. While numerous models of smart home services exist, there is a lack of applicable theoretical frameworks for modeling smart home services for seniors. To address the specific needs of the older adult, we focused on the primary design guidelines found in past literature and extracted a smart home model that is more suitable for their well-being (10, 12, 15). Figure 2 illustrates the model’s five dimensions: Environment Monitoring (EM), Health Monitoring (HM), Community Management (CM), Amenity Improvement (AI), and Risk Management (RM). For the sake of user comprehension, the model omits the technical description of information collection and transmission.

Due to the tendency of seniors to rely on heuristics when making decisions (64), additional questions were included in the survey to trigger semantic processing. To facilitate the comprehension and recollection of smart home systems and devices used in their lives, we presented each of the five dimensions of the smart home model in the form of questions and asked respondents to rate the usefulness of five devices using a seven-point Likert scale. Previous studies have demonstrated the effectiveness of similar methods (65, 66). As most of these studies focus on users over the age of 55 (14), we also restricted our study to this age group. Before distributing the questionnaire to the target population, we consulted two independent experts in the field of user experience to check the validity and consistency of the developed questionnaire. We used a convenience sampling method, creating a link to the questionnaire on the website “Wen Juan Xing” and distributing it through several WeChat chat groups. Older Adult people in the groups completed the questionnaire directly, while younger members of the groups sent the link to their senior relatives to complete the survey.

As technological products become more prevalent in homes, an increasing number of senior citizens are adopting smart home technology. However, due to their inexperience with smart home technology, many older adult individuals are not able to fully utilize these systems. Furthermore, the stage of adoption can affect the level of technology acceptance and influencing factors among older adults who use smart home services (20, 47, 67). To ensure the accuracy of the data, we screened senior adults who had used smart home products using a series of questions designed to reduce hypothetical answer bias among participants who had never used a smart home. Specifically, we included a screening question: “Have you ever used smart home technology/services?” Response options included “often,” “occasionally,” “not sure if I have used it,” and “have not used.” Users who answered “not sure if I have used it” and “have not used” were filtered out and did not continue with the survey.

We measured intergenerational emotional support through three questions, adapted from the Intergenerational Solidarity Survey (68). Since intergenerational financial support includes gift-giving (62), we asked the respondents if they had received any monetary or gift support from their children in the 2 years prior to the survey in order to determine the level of intergenerational financial support. Considering the high cost of smart home technology and its prevalent use in urban areas, this study attempted to distribute questionnaires mainly among the older adult population residing in urban areas, in order to seek a larger pool of respondents who have used smart home technology. Responses to the questionnaire were rated on a five-point Likert scale ranging from 1 (strongly disagree) to 5 (strongly agree). Table 1 presents the final variable definitions and their corresponding sources.

**Table 1:** Construct operationalization along with descriptive statistics.

Measured variables	Measured question	Items	Content source
Perceived usefulness (PU)	PU1	Using smart home will make my life more convenient	Davis et al. (44) Moore and Benbasat (69)
	PU2	Using smart home can make my life more independent and secure	
	PU3	Using smart home, my life will become more enjoyable	
Perceived ease of use (PEOU)	PEOU1	I think the smart home system is easy to use	Davis et al. (44)
	PEOU2	My interaction with the smart home system is simple and clear	
	PEOU3	I can easily learn how to operate the smart home products	
Intergenerational instrumental support (IIS)	IIS1	My children (or grandchildren) have provided me with electronic products	Lang and Schütze (70) He and Huang (40)
	IIS2	My children (or grandchildren) have encouraged me to use electronics (or helped me set up electronics)	
	IIS3	My children (or grandchildren) help me when I have trouble using electronics I get along well with my children	
Intergenerational emotional support (IES)	IES1	I get along well with my children	Mangen et al. (68) Chen and Chan (14)
	IES2	My children are willing to listen when I talk about my concerns and problems	
	IES3	My children and I are close	
Intergenerational financial support (IFS)	IFS1	My children have supported me financially (living expenses, money, etc.) in the past two years	Chang and Huang (55) Chen and Chan (14)
	IFS2	My children have given me money, food, or gifts in the past two years, and they are worth a lot of money	
Behavioral intention (BI)	BI1	If I had a smart home system, I would use it	Davis et al. (44)
	BI2	I have an interest in using a smart home	
	BI3	I predict that I will use more smart homes in the future	
Life satisfaction (LS)	LS1	In most respects, my life is close to ideal	Diener et al. (71)
	LS2	My living conditions are very good	
	LS3	I am very satisfied with my life	

## Model analysis and results

A total of 298 valid questionnaires were collected in this study, with 128 (43%) males and 170 (57%) females responding. The respondents’ basic demographic information is presented in Table 2. SPSS20 and Smart PLS3.0 were used to analyze and statistically process the data, with the PLS-SEM technique utilized since it is optimal for exploratory investigations.

**Table 2:** Demographic of respondents.

Profile	Sample composition	Frequency	Percentage
Gender	Male	128	42.95
	Female	170	57.05
Age	56–60	207	69.46
	61–65	57	19.13
	66–70	19	6.38
	Over 71	15	5.03
Education background	Primary education	39	13.09
	Junior high school or equivalent	65	21.81
	Senior high school or equivalent	107	35.91
	College degree or above	87	29.19
Monthly Income	Less than 4,000	150	50.34
	4,000–7,000	106	35.57
	7,000–10,000	25	8.39
	10,000–15,000	10	3.36
	More than 15,000	7	2.35

To examine the impact of controlling variables on the relationship between the independent variable and the dependent variable, we assessed the model both with and without controlling variables. The findings demonstrated that the difference in standardized coefficients of the independent variable between the two models was less than 0.1. As advised by Becker et al. (72), this suggests that the effects of controlling variables are negligible, and therefore, we presented only the results without controlling variables in the subsequent section.

The collected data were analyzed using Confirmatory Factor Analysis (CFA) and Structural Equation Modeling (SEM) methods. First, a CFA was performed on eight constructs (dimensions), as shown in Table 3. For all the constructs used, Cronbach’s alpha values obtained were more significant than 0.7 and synthetic reliability (CR) greater than 0.6, which indicates a high degree of internal consistency (73). For each construct, the Average Variance Extracted (AVE) is more significant than 0.5, indicating good convergent validity of the measurement model (74). According to Henseler et al. (75), all the values in the HTMT must be less than 0.90. As presented in Table 4. The results indicate that these constructs fulfill the discriminant validity.

**Table 3:** Standardized factor loadings, CRs and AVEs and Cronbach’s alphas.

Construct	Item	Factor loading	Cronbach’s alpha	rho_A	Composite reliability	AVE
Behavioral intention	BI1	0.913	0.905	0.905	0.941	0.841
	BI2	0.938				
	BI3	0.899				
Intergenerational instrumental support	IIS1	0.849	0.833	0.838	0.900	0.749
	IIS2	0.881				
	IIS3	0.866				
Intergenerational emotional support	IES1	0.910	0.858	0.871	0.914	0.779
	IES2	0.905				
	IES3	0.831				
Perceived ease of use	PEOU1	0.891	0.888	0.890	0.931	0.818
	PEOU2	0.910				
	PEOU3	0.912				
Perceived usefulness	PU1	0.848	0.839	0.841	0.903	0.757
	PU2	0.872				
	PU3	0.890				
Life satisfaction	LS1	0.918	0.859	0.869	0.915	0.782
	LS2	0.819				
	LS3	0.912				
Intergenerational financial support	IFS1	0.793	0.703	0.879	0.861	0.758
	IFS2	0.942				

**Table 4:** Heterotrait-monotrait tatio (HTMT).

	IIS	IES	PEOU	PU	LS	IFS	BI
IIS							
IES	0.801						
PEOU	0.547	0.477					
PU	0.699	0.663	0.77				
LS	0.57	0.588	0.391	0.487			
IFS	0.47	0.424	0.359	0.385	0.524		
BI	0.527	0.432	0.567	0.588	0.436	0.221	

To assess the absence of correlation between measurements, divergent validity was examined. Table 5 displays the obtained results. The diagonal element, which represents the square root of AVE, has a higher correlation level between any two specific factors. As a result, the vast majority of constructs in this study exhibit good discriminant validity (76).

**Table 5:** Correlation matrix among constructs and square root of AVEs.

	BI	IIS	IES	PEOU	PU	LS	IFS
BI	0.917						
IIS	0.458	0.864					
IES	0.383	0.684	0.883				
PEOU	0.509	0.471	0.419	0.904			
PU	0.514	0.583	0.564	0.665	0.870		
LS	0.382	0.482	0.510	0.343	0.414	0.884	
IFS	0.187	0.360	0.322	0.298	0.307	0.440	0.870

Tenenhaus et al. (77) has presented an alternative method for determining the goodness of fit (GoF). The formula for the GoF value is as follows:

$$\mathrm{GoF}=\sqrt{\bar{\mathrm{AVE}}\times \bar{R^{2}}}=\sqrt{0.783\times 0.426}=0.578.$$

We obtain a GoF value of 0.578, which is greater than the suggested GoF value of 0.36 (77), proving the validity of the model.

The hypothesis testing was done in Smart PLS 3.0. We used the bootstrapping method (78). According to the criteria of Henseler et al. (79), SRMR <0.08 is acceptable, we derived an SRMR of 0.061.

**Figure 3:**
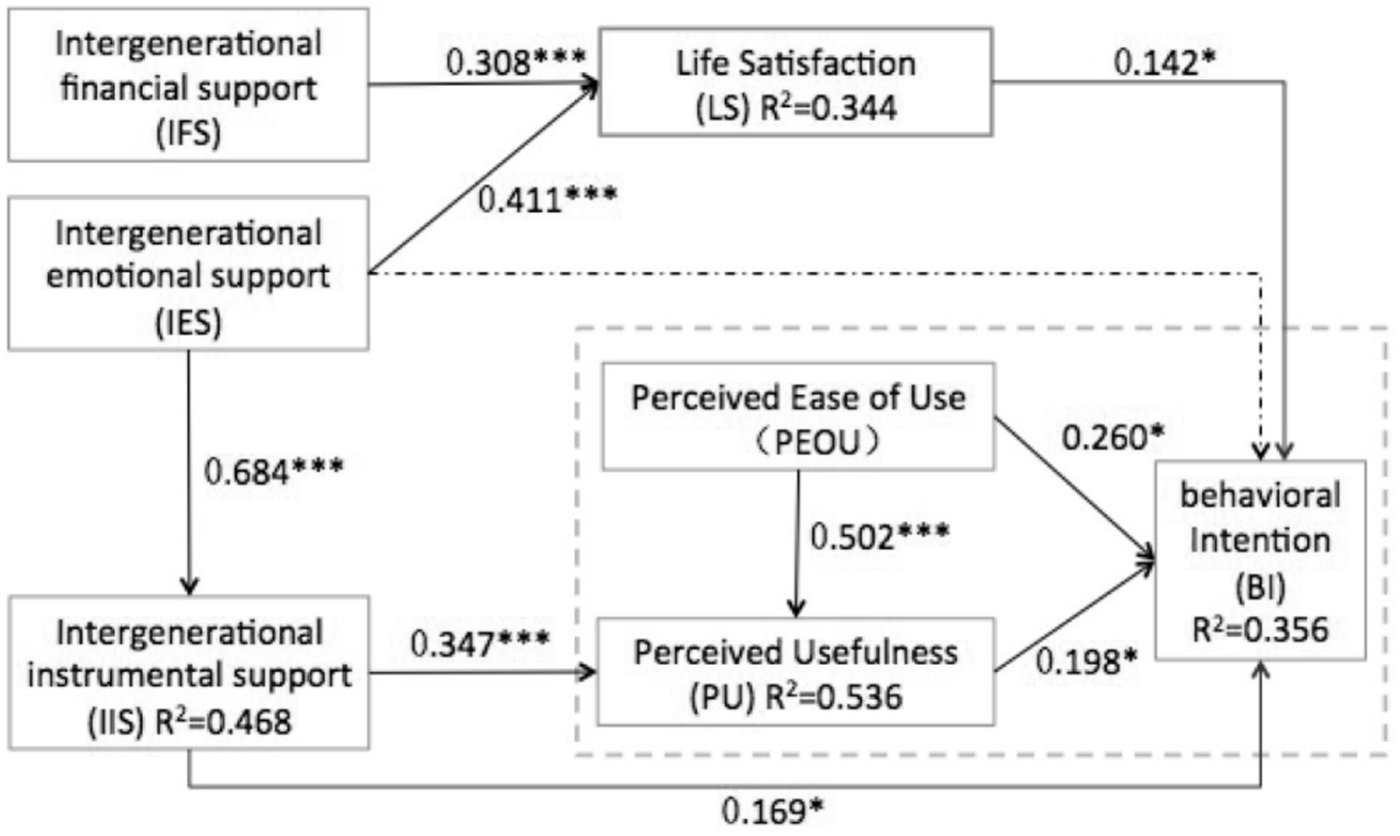
Final theoretical framework. *p < 0.05, ***p < 0.001. One dotted line indication non-significant path was added in making all proposed factors shown in an integral model.

The subsamples were randomly selected from the original data set, and this process was repeated multiple times to create a large number of random subsamples. The results indicate that all hypotheses are supported, except for H7. Table 6 presents the significant results of each path coefficient, and the final theoretical framework is illustrated in Figure 3.

**Table 6:** Results of path analysis and hypotheses testing.

Hypothesis	Standardized coefficient (β)	*t*-Statistics	*p*-Value	Hypothesis status
PU → BI	0.198	2.326	0.020*	Supported
PEOU→BI	0.260	3.376	0.001*	Supported
PEOU→PU	0.502	9.832	<0.001***	Supported
IIS → PU	0.347	7.226	<0.001***	Supported
IIS → BI	0.169	2.166	0.030*	Supported
IES → LS	0.411	6.808	<0.001***	Supported
IES → BI	−0.025	0.345	0.730	Not supported
IES → IIS	0.684	15.631	<0.001***	Supported
LS → BI	0.142	2.281	0.023*	Supported
IFS → LS	0.308	5.150	<0.001***	Supported

*Significant at *p* < 0.05, ***significant at *p* < 0.001.

## Discussion

The findings of this study suggest that many of the explanatory variables are highly significant, and even after using technology, the Technology Acceptance Model (TAM) still has explanatory power regarding older adults’ acceptance of smart homes. PEOU has a significant positive effect on both PU and BI. Although PU also profoundly influenced BI, PEOU (standardized path coefficient of 0.260) had a greater effect on BI than PU (standardized path coefficient of 0.198). This supports previous research indicating a strong link between perceived ease of use and technology acceptance among older adults (80). These findings emphasize the importance of making smart home technology simple and easy to use to meet the requirements of senior citizens, especially by designing appropriate voice interface styles, interface navigation, swipe layout, and button size based on the cognitive behavior of older users (81, 82).

However, Pal et al. (15) found the opposite result in their study of smart home use by older adults, concluding that the influence of usefulness on behavioral intentions is more significant. One possible explanation for this discrepancy could be gender differences; Venkatesh and Morris (83) suggest that the effect of perceived usefulness on intention should be stronger for older adult men than for older adult women. In Pal’s study, men comprised 65.7% of the participants, while in this study, men only comprised 42.95%.

Consistent with Peek’s earlier findings (19), our study reveals that although older adults are aware of the advantages of smart homes in terms of increased independence and safety, this awareness does not necessarily translate into a willingness to use them. This may be because many older adult people believe that smart home technology is mainly aimed at older adults who are in poorer health conditions, rather than themselves. Additionally, other barriers may significantly hinder the perceived benefits of using smart homes.

### Intergenerational support

Our research suggests that intergenerational support plays a critical role in facilitating the use of smart home services by older adults. Specifically, three types of intergenerational support directly or indirectly influence the usage of smart home services by the older adult. Firstly, the provision of home electronics to older adults, along with training them on the use of technology, can directly enhance their perception of the usefulness of smart home services. Prior research supports this positive effect (51). We believe that intergenerational tools used by children can also aid older adults in connecting with smart home technology. Once older adults experience the practical benefits of smart home products, a positive cycle will be generated, ultimately eliminating apprehensions about unfamiliar technology and resulting in the acceptance of additional smart home services.

Numerous studies have indicated the significance of the social relationships of older adults, including support and guidance from family and friends, in the adoption of technology (14, 17, 33). Due to the importance of family bonds in traditional Chinese culture, many citizens prefer the three-in-a-row model where parents and children provide intergenerational upward and downward support. Therefore, it is crucial to concentrate on the relationship between older adults and their children rather than other relationships when studying the use of smart home technology. This study found that both emotional and financial support predict the intention to use smart homes through life satisfaction. In particular, older adults who received more emotional and economic support from their children reported higher life satisfaction and were therefore more inclined to use smart home services.

An active lifestyle can increase users’ willingness to learn new technologies (14). As hypothesized, this study demonstrated that life satisfaction is positively correlated with the willingness to use smart homes among older adults. This suggests that positive emotions enable older adults to manage complex technologies better and be more open to new technological challenges. Boosting their confidence in their ability to use technology allows seniors to establish connections to smart home services more quickly. Since the use of smart-home devices is argued to enhance the well-being of older adults (5, 58), the relationship between life satisfaction and the use of smart homes may be interactive. The greater satisfaction with life, the more likely it is that smart home technology will be used, and this, in turn, may enhance the well-being of older adult users. Ren and Klausen (84) contend that society should encourage the older adult to utilize cell phones more frequently to enhance their sense of well-being. The study by Wu and Chiou (85) suggests that social media use among older adults can effectively improve intergenerational relationships and alleviate depressive symptoms. Building on this research, we propose that increased use of smart home services can also enhance the well-being of older adults, in turn, can lead to a more extensive utilization of smart home services and facilitating aging in place.

Consistent with the findings of Silverstein and Bengtson (56), our study found that intergenerational emotional support provides greater life satisfaction to older adult individuals than intergenerational financial support. Notably, when intergenerational instrumental support was used as a mediator, the indirect effect of intergenerational emotional support on behavioral intentions was significant. However, there was no direct effect between the two. This suggests that emotional support between parents and children is an important factor. Although emotional support cannot directly influence parents’ intention to use smart home services, it can facilitate the intergenerational transfer of technological knowledge, ultimately helping to bridge the digital divide among older adult individuals.

Therefore, when promoting smart home products in community homes to enhance the independence of older adults, more emphasis can be placed on the perspective of intergenerational support. For instance, to alleviate negative feelings towards smart homes among older adult individuals, children can present them with necessary products and instructions on how to use them. As mentioned in previous empirical studies, many parents emphasized the importance of their children’s concerns when deciding whether they needed a service or technology (86).

When using age and gender as control variables, this study found that these variables did not have a significant impact on the model, indicating that older adults’ attitudes towards smart homes may be influenced by multiple complex factors. The decline in cognitive ability associated with aging may hinder the acceptance of older adult technology (49), while the decline in health may enhance their acceptance of technology products, as they may view technology as a means of compensating for and facilitating independent living (14). These age-related factors may have either positive or negative effects on the older adult’s acceptance of smart homes, contributing to the lack of correlation between age and smart home acceptance. Further research is needed to investigate these factors in greater detail.

## Conclusion and limitations

Smart home technology is considered an effective means of supporting in-home aging for seniors, as it can significantly improve their health and independence. However, despite increasing attention, smart homes are not widely adopted among the older adult. Therefore, when developing and delivering smart home technology for older adults, it is important to understand the social supports that influence their use and decision-making, as well as to fully comprehend their needs and provide solutions that are easier to use.

The influence from their family can overcome the fear and rejection of older adults towards smart home technology. This paper contributes to a better understanding of the factors influencing in the initial acceptance of smart home technology by older adults. When seniors live with their children, they have access to a wide variety of intergenerational support, which can have a positive external effect on them. In Chinese households, which tend to consist of extended families, older adults’ acceptance of new technologies may be significantly enhanced. Public policies that incentivize intergenerational interactions can help achieve this goal.

Future research can continue to explore related directions, such as how family relationships affect the number of smart home devices used by the older adult, how to increase their interest in using them, and how to improve their skills. However, the technical maturity of smart home services is currently low and most studies have focused on health monitoring technologies, limiting the possibility of studying the acceptance factors of smart homes after full use.

There are several limitations to this study. First, although the participants were randomly selected, some selection bias may have occurred. For example, many of our questionnaires are delivered to older adult people through their children, so older adult people with high emotional cohesion with their children are more likely to receive this questionnaire that we have sent out. Additionally, the age range of the participants was relatively young, which may not fully represent the intergenerational support and acceptance of smart home services among the older adult population. Hence, the results need to be interpreted with caution. Second, this study provides a general understanding of smart home acceptance. However, there are various categories of smart homes with different levels of usability, ease of use, and purpose, which may influence older adults’ attitudes towards them. Therefore, future research should segment the study based on different types of smart homes. Third, the study does not consider the impact of brand influence on older adults’ attitudes towards smart homes, as different brands of smart home products have different interfaces and designs. Therefore, future research should take brand influence into account. In conclusion, more research is needed to capture the complexity of the acceptance process of different types of smart home services by older adults in the community to better leverage technology for their aging-in-place.

## Data availability statement

The original contributions presented in the study are included in the article/supplementary material, further inquiries can be directed to the corresponding author.

## Author contributions

WW proposed the idea of this research, wrote the paper, and acquired the funding. XG wrote a part of the paper. KT, JL, and KX collected the data and revised the paper. All authors contributed to the article and approved the submitted version.

## Funding

This work was supported by the key project of the Beijing Social Science Foundation funded by the Beijing Federation of Social Sciences (18YTA001).

## Conflict of interest

The authors declare that the research was conducted in the absence of any commercial or financial relationships that could be construed as a potential conflict of interest.

## Publisher’s note

All claims expressed in this article are solely those of the authors and do not necessarily represent those of their affiliated organizations, or those of the publisher, the editors and the reviewers. Any product that may be evaluated in this article, or claim that may be made by its manufacturer, is not guaranteed or endorsed by the publisher.
